# Stability of Tin-Containing Hybrid Perovskites: The Thermal Decomposition of Formamidinium Tin Triiodide (FASnI_3_) Investigated by Thermogravimetry and Effusion Techniques

**DOI:** 10.1021/acs.jpcc.5c01854

**Published:** 2025-05-10

**Authors:** Martina Pesci, Lorenza Romagnoli, Bruno Brunetti, Stefano Vecchio Ciprioti, Andrea Ciccioli, Alessandro Latini

**Affiliations:** 1 Dipartimento di Chimica, 154900 Sapienza University of Rome, P.le A. Moro 5, Rome 00185, Italy; 2 Istituto per lo Studio dei Materiali Nanostrutturati, Consiglio Nazionale delle Ricerche, Dipartimento di Chimica, 154900 Sapienza University of Rome, P.le A. Moro 5, Rome 00185, Italy; 3 Dipartimento di Scienze di Base ed Applicate per l’Ingegneria (S.B.A.I.), Sapienza University of Rome, Via del Castro Laurenziano 7, Building RM017, Rome 00161, Italy

## Abstract

The gas-releasing thermal decomposition processes of formamidinium tin triiodide perovskite (CN_2_H_5_SnI_3_, usually denoted as FASnI_3_ or, briefly, FASI) were investigated in order to obtain information on the temperature at which decomposition begins and on the nature of the gas species emitted under heating. Results of thermogravimetry-differential thermal analysis (TG-DTA), thermogravimetry-differential scanning calorimetry (TG-DSC), and Knudsen effusion mass spectrometry (KEMS) experiments are presented. TG measurements showed that mass loss starts at temperatures much lower than those of the lead-based corresponding compound FAPbI_3_, with incipient loss at temperatures as low as 423 K. Unlike FAPbI_3_, practically no residue is left at *T* = 823 K. KEMS experiments showed a measurable release of SnI_4_(g) occurring from temperatures as low as 318 K, with a SnI_4_(g) pressure much lower than the vapor pressure of pure SnI_4_, suggesting the presence in the solid of Sn­(IV) at reduced activity. At higher temperatures (*T* > 390 K), the release of SnI_2_(g) and various species coming from the organic portion, such as formamidine, hydrogen iodide, ammonia, triazine, and hydrogen cyanide, is observed. For the first time, thermal decomposition of FASI was shown to occur with release of both organic and inorganic (tin iodides) species, with the partial oxidation of Sn­(II) to Sn­(IV) significantly decreasing the decomposition onset temperature. Finally, based on TG measurements at various scan rates, a kinetic analysis of FASI decomposition was performed, using integral and incremental isoconversional methods, to derive parameters useful for lifetime prediction. Reasonable reaction time values to achieve a low degree of conversion (less than 0.25) were extrapolated at temperatures typically involved in the operation of photovoltaic devices (333 to 353 K).

## Graphical abstract

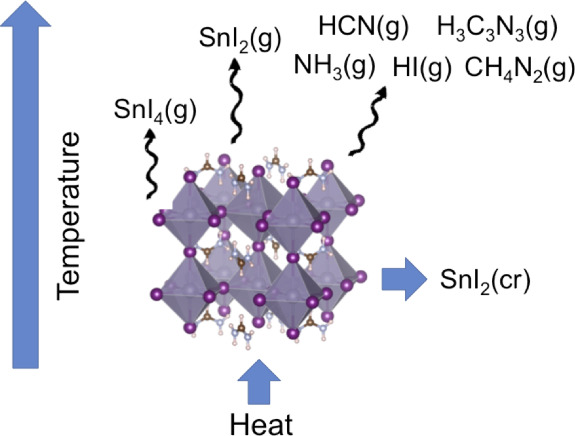

## 1 Introduction

While the need to reduce CO_2_ emissions, in order to realize the goal of net zero emissions by 2050, is increasingly urgent, solar photovoltaic electricity generation is expected to surpass hydropower and become the largest renewable power source in 2029, according to the International Energy Agency forecasts.^1^ This implies, among other things, reducing costs for photovoltaic cell materials and manufacturing, to make solar energy exploitation affordable worldwide. Although silicon-based solar panels have become increasingly cheaper in the last decades, thanks especially to the introduction of polycrystalline and amorphous Si, nevertheless the need for extremely high purity and doping still prompts research for new, inexpensive, and easily manufactured semiconductors.

Among the multitude of currently investigated new generation solar cells (so-called third-generation cells, where the first generation is represented by wafer-based and the second by thin-film ones^2^), perovskite solar cells (PSC) have captivated an unprecedented attention, since the first reported example, in 2009, of a solar cell containing hybrid perovskites, CH_3_NH_3_PbX_3_ (where X = Br, I).^3^ This is due to the extraordinary properties of these semiconductors, which possess not only band gap values in the suitable range for photovoltaics, but also low exciton binding energies, high absorption coefficients, and long charge carrier diffusion lengths.^4^ Moreover, perovskites offer very attractive prospects also in terms of material synthesis, since they can be conveniently obtained, as bulk crystals, nanocrystals, and thin films, by various low-temperature solution techniques,^5^ in contrast to many other semiconductors.

The other side of the coin is unfortunately represented by commercialization setbacks caused by poor long-term stability of hybrid perovskites, due to heating and to the effect of environmental agents such as water, oxygen, and UV radiation,^6,7^ and by health and environmental concerns related to the presence of lead.^8^ Nevertheless, various alternatives to lead have recently gained increasing consideration:^9^ for example, Ge- and Sn-based halide perovskites, of general formula ABX_3_, Sb- and Bi-based halide perovskites, of general formula A_3_B_2_X_9_, and chalcogenide (especially sulfur-containing) perovskites, having general formula ABCh_3_ (Ch = S, Se). Among all these, however, only tin halide perovskites have hitherto shown comparable (although still inferior) performances and viability to their lead counterparts for solar cells: indeed, the highest certified power conversion efficiency (PCE) on a laboratory scale for a Pb-containing PSC is 26.7%,^10^ while the best reported value for Sn-based ones is 15.7%;^11^ by contrast, the highest achieved efficiency with bismuth-based perovskites is currently only 3.59%.^12^

The assessment of halide perovskites as really promising candidates for large-scale distribution needs to take into account the possible sources of their detriment under operating conditions. In this regard, great efforts have been conducted in recent years to understand the different facets of lead perovskite instability, for example the effect of heat and light irradiation^13,14^ and the thermodynamic^15–17^ and kinetic aspects^18,19^ of their thermal decomposition.

However, as concerns tin-based perovskites, oxidation of Sn^2+^ to Sn^4+^ is known to constitute a serious issue, causing the formation of undesired vacancies, leading in turn to a self-doping of the material, which makes control of electric properties difficult.^20,21^ Although numerous strategies and additives have been devised to mitigate this trouble,^22,23^ the problems seem to be inherently unavoidable.^24^ Moreover, in contrast to the already mentioned profusion of studies regarding decomposition of lead halide perovskites and despite some recent progress in the comprehension of tin halide perovskite stability,^25–27^ information about the degradation mechanisms of the latter is still comparatively limited.

Our work lies in this context and is specifically devoted to investigating the role of Sn­(II) → Sn­(IV) oxidation in decreasing the thermal stability of formamidinium tin triiodide (CN_2_H_5_SnI_3_, FASI in the following), one of the most widely investigated Sn-based perovskites for application in solar cells^28^ thanks to the lower volatility of the organic cation, compared to methylammonium. More generally, this study was aimed at elucidating the thermal decomposition pathways of FASI, which were found to be much different from those of the Pb-containing analogue (CN_2_H_5_PbI_3_, FAPI in the following). A multitechnique approach, combining effusion techniques, in particular Knudsen effusion mass spectrometry (KEMS), with thermal analysis measurements, was used in this work, similar to that applied by our group to the corresponding Pb-containing material.^29^ KEMS measurements allowed the assessment of the gaseous products released from the perovskite upon heating at moderate temperatures, under close-to-equilibrium conditions, and the estimation of partial pressures of the decomposition products. To this end, ancillary experiments were also carried out on binary iodides, SnI_2_ and SnI_4_. Thermogravimetric analysis of the perovskite and tin iodides, on the other hand, allowed us to determine the onset and the various steps of thermal degradation. Furthermore, by performing TG-DTA at different heating rates and applying the isoconversional method, an overall value for the activation energy of the decomposition reactions was derived.

## 2 Methods

### 2.1 Synthesis

All of the reagents were used as received. Tin powder (99.8%) and formamidinium iodide (≥98%) were supplied by Sigma-Aldrich, iodine (99.9%) was purchased from BDH Chemicals, and CH_2_Cl_2_ (for analysis, ≥99.9%) and HCl (analytical grade, 37%) were supplied by Carlo Erba Reagents.

#### 2.1.1 Synthesis of SnI_2_

SnI_2_ was prepared according to a modified literature procedure,^30^ by reacting in a round-bottomed flask 5 g of Sn powder with 7 g of I_2_ in 30 mL of a 2 M HCl solution, under an argon atmosphere. The suspension was heated to reflux for 2 h until a yellow solution was obtained; then, it was filtered to remove excess Sn and transferred into a conical flask, warmed by a hot water bath. The solution was allowed to cool overnight to room temperature, to facilitate crystallization of the product, and then SnI_2_ red crystals were filtered under suction, washed with a degassed 0.02 M HCl solution, and dried at 323 K.

#### 2.1.2 Synthesis of FASnI_3_

Formamidinium tin triiodide (CN_2_H_5_SnI_3_) was synthesized, following a literature procedure,^31^ by a solid-state reaction between commercially available formamidinium iodide (CN_2_H_5_I) and SnI_2_: equimolar amounts of the reagents (400 mg of SnI_2_ and 194 mg of CN_2_H_5_I) were ground in an agate mortar for 5 min, until a black powder was formed; the product was inserted in a glass ampule, subsequently evacuated with a rotary vane pump to ∼10^–1^ mbar, flame sealed, and then transferred in an oven where it was heated for 2 h at 473 K.

#### 2.1.3 Synthesis of SnI_4_

For the synthesis of SnI_4_, 6 g of Sn powder and 20 g of I_2_ were placed in a round-bottomed flask with 40 mL of CH_2_Cl_2_, and the mixture was heated to reflux for 1 h until the color turned from purple to yellow-orange (indicating the disappearance of I_2_), and then quickly filtered while still warm, to remove unreacted tin. The filtered solution was cooled through an ice bath to allow crystallization of the product, and SnI_4_ dark-orange crystals were filtered under suction, washed with cold CH_2_Cl_2_, and dried in the air.

The identity and purity of the compounds were assessed by powder XRD. All of the compounds were obtained in pure crystalline form and used as synthesized, without further purification.

### 2.2 Powder X-ray Diffraction

Powder X-ray diffraction patterns, in the 20–90° 2θ angular range, of the as-synthesized samples and of CN_2_H_5_SnI_3_ residue after KEMS experiments were acquired with a Malvern Panalytical X’Pert Pro MPD diffractometer (Cu Kα radiation, λ = 1.54184 Å), operating in Bragg–Brentano geometry and equipped with an ultrafast X’Celerator RTMS detector. The experiments undertaken in the attempt to study the kinetics of nucleation of SnI_2_ (see Section 3.2) were conducted according to the procedure described in ref 29.

### 2.3 Thermogravimetry-Differential Scanning Calorimetry

Thermogravimetry-differential scanning calorimetry experiments on CN_2_H_5_SnI_3_, SnI_2_, and SnI_4_ were performed with a NEXTA STA200RV Hitachi simultaneous thermal analyzer in alumina crucibles, in the temperature range 298–1073 K, with a heating rate of 10 K·min^–1^. The analyses were carried out on 10 to 20 mg powder samples under an inert atmosphere (150 mL·min^–1^ @ STP Ar for CN_2_H_5_SnI_3_, 100 mL·min^–1^ @ STP N_2_ for SnI_2_ and SnI_4_), and the samples were conditioned for 30 min in the same conditions prior to heating, to avoid oxidation.

### 2.4 Thermogravimetry-Differential Thermal Analysis

Thermogravimetry-differential thermal analysis measurements of CN_2_H_5_SnI_3_ for kinetic analysis through the isoconversional method were performed with a Netzsch STA 409 PC Luxx simultaneous thermal analyzer, in alumina crucibles, in the temperature range 298–773 K, under flowing Ar atmosphere (50 mL·min^–1^ @ STP), using sample mass of about 40 mg, precisely measured. After the sample was weighed, the measurement chamber was quickly evacuated with a rotary vane pump down to a pressure of around 10^–2^ mbar and then refilled with Ar (purity ≥ 99.9995%) up to atmospheric pressure. This procedure was performed to avoid the oxidation of the sample. The heating rates used were 2, 3, 4, 7, and 10 K·min^–1^.

### 2.5 KEMS and Knudsen Effusion Mass Loss (KEML)

In these techniques, the sample is placed, under high vacuum, in a cylindrical cell equipped with a lid in which a small hole is drilled, allowing the vapor phase to escape under the molecular flow regime.

In KEMS, the vapor effusing from the cell enters the ion source of a mass spectrometer, where neutral species are positively ionized by electron impact, accelerated, separated according to their mass to charge ratio, and finally revealed. In our apparatus,^15,29^ a single focusing magnetic sector mass spectrometer is used and ion currents are measured by a secondary electron multiplier. Ions coming from the effusion cell are distinguished from background species by means of a movable shutter placed between the cell and the ion source. Ion intensities, from which partial pressures are obtained (see eq 1 below), are taken as the difference between the total intensity and that measured when the shutter is shifted.

In the experiments described here, a graphite cell with a 1 mm orifice in the lid (also made of graphite) was used. The effusion cell was enclosed in an outer tantalum crucible and was surrounded by a spiral-shaped tungsten heating element. Finally, the crucible and heating coil are surrounded by several tantalum thermal shields. Temperature measurement was carried out through a Pt10%Rh–Pt (type S) thermocouple, placed in a hole made in the bottom of the external tantalum crucible. Ionization energy curves and appearance energies, i.e., the minimum electron energy necessary to produce a given ion, were measured for all of the detected ions. Appearance energies were determined by using the vanishing current method and corrected based on the ionization energy of water,^32^ taken as a reference. The energy of the ionizing electron beam was adjusted according to the maximum ionization efficiency of each ion and was in the range 17–24 eV. Partial pressures of the neutral species *i* in the vapor were calculated from the intensities of the ions produced there from, by eq 1:

$$P_{i}=\frac{k_{\mathrm{instr}}}{\sigma_{i}}\underset{k}{\sum }\frac{I_{k}^{+}T\sqrt{M_{k}}}{a_{k}}$$ 1

In this equation, *I*_*k*_^+^ represents the ion current of the ion species *k* (molecular ion or fragment) formed from *i* under electron impact, *M*_*k*_ is the molecular mass, *a*_*k*_ is the abundance of the observed isotope of the *i* species, σ_*i*_ corresponds to the total ionization cross section, *T* is the absolute temperature, and *k*_instr_ is an instrumental constant, corresponding to $\frac{P_{\mathrm{ref}}a_{\mathrm{ref}}\sigma_{\mathrm{ref}}}{I_{\mathrm{ref}}^{+}\sqrt{M_{\mathrm{ref}}}T_{\mathrm{ref}}}$ , obtained through calibration experiments with a substance of known vapor pressure. Calibration was carried out by vaporizing, in the lower and higher temperature ranges of our experiments, respectively, 1,3,5-triphenylbenzene (powder, 99.9%) and zinc (pieces, 99.9%), both supplied by Sigma-Aldrich.

KEML measurements were carried out with the apparatus described in ref 33. An effusion cell in pyrophyllite was used with a diameter of the effusion hole of 1 mm. In this method, the measured mass loss is related to total vapor pressure *P* through eq 2:

$$P=K\frac{dm}{dt}\sqrt{\frac{2\pi RT}{\overset{¯}{M}}}$$ 2

where *K* is a constant depending on the geometrical characteristics of the effusion hole, $\frac{dm}{dt}$ is the rate of mass loss, *T* is the absolute temperature, and *M̅* is the average molar mass of the vapor, calculated by eq 3, where *x*_*i*_ is the mole fraction of the *i* species and *M*_*i*_ its molecular mass:

$$\mathrm{M̅}={\left(\underset{i}{\sum }x_{i}\sqrt{M_{i}}\right)}^{2}$$ 3

In this work, KEML was used only to verify if the total pressures were or were not constant at a given temperature.

## 3 Results

### 3.1 TG-DSC of CN_2_H_5_SnI_3_, SnI_2_, and SnI_4_

The thermal behavior of FASI was assessed by TG-DSC (Figure 1), performed under an inert atmosphere from room temperature to 1073 K.

**Figure 1:**
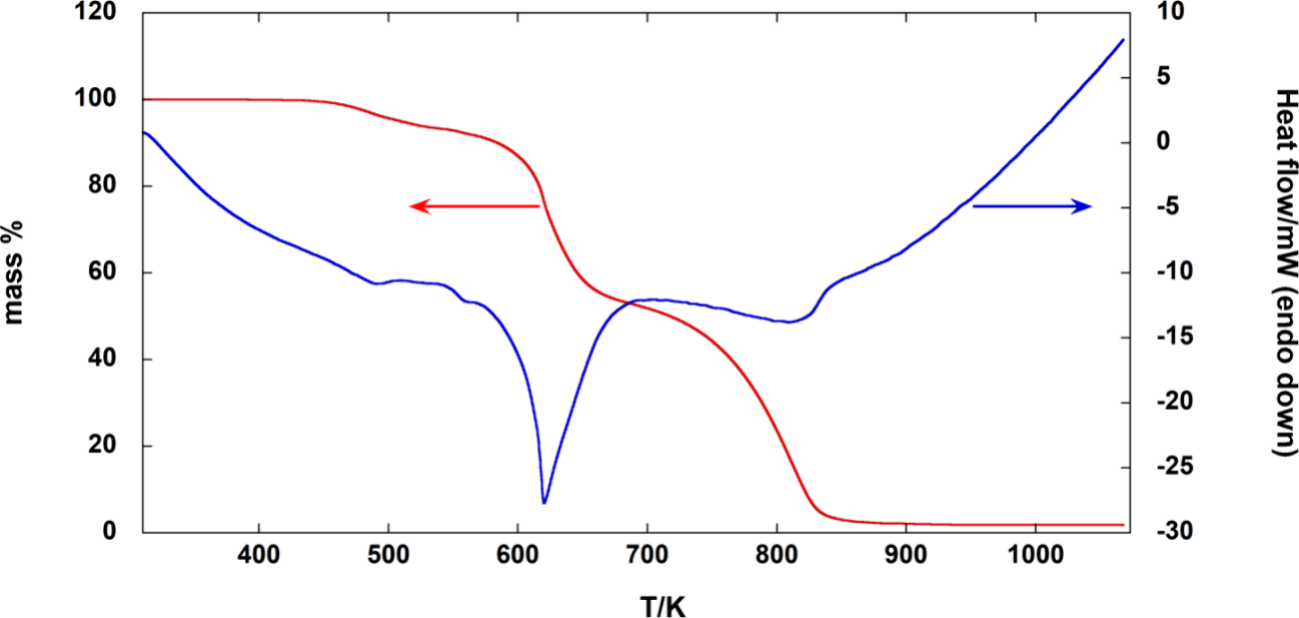
TG-DSC curve of CN_2_H_5_SnI_3_ carried out under an Ar atmosphere (150 mL·min^–1^) at 10 K·min^–1^.

The TG curve shows an initial decomposition step, involving about 6.7% mass loss, taking place between 420 and 550 K, followed by a steeper decrease in mass (around −40%) up to about 700 K, characterized by a large endothermic peak. These sequential mass loss steps reproduce qualitatively the behavior already reported in previous TG data from the literature.^31,34–36^ Finally, practically all the remaining material is converted into volatile species at 850 K, leaving about 1.7% residue at 1073 K. This thermogravimetric curve can be compared to that of the corresponding lead perovskite.^29^ As shown in Figure 2, there is a striking difference between the CN_2_H_5_SnI_3_ and CN_2_H_5_PbI_3_ thermal behavior, with loss of volatile decomposition products from the latter starting at much higher temperatures, around 570 K. Moreover, the residual mass of 72.8% at 773 K in CN_2_H_5_PbI_3_, corresponding to the PbI_2_ mass fraction, proves that no Pb-containing gaseous decomposition products are formed below this temperature. By contrast, more than 40% mass of CN_2_H_5_SnI_3_ is lost below 700 K, while the CN_2_H_5_I mass fraction is only 32%, pointing to the formation of volatile Sn-containing degradation products in this temperature range. Although the experimental conditions used in TG and KEMS measurements are very different (higher *T* range in TG, open pan *vs* Knudsen cell, inert atmosphere *vs* high vacuum), an attempt can be done to explain the observed TG mass loss behavior in light of KEMS findings (see Section 3.3). On this basis, it can be supposed that the thermal behavior below 550 K is due to the release of volatile Sn­(IV) species, whereas the mass loss up to about 700 K can be ascribed to the simultaneous evolution of gaseous decomposition products from the organic portion and sublimation of SnI_2_ formed as a result of these degradation processes (see eqs 9–11 in Section 4), as mentioned above.

**Figure 2:**
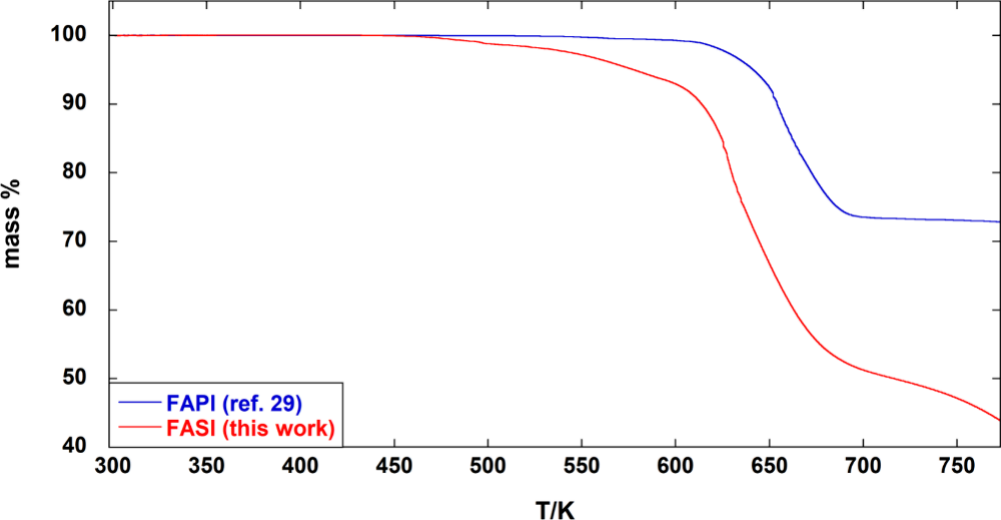
Comparison between TG curves of CN_2_H_5_PbI_3_ (FAPI) and CN_2_H_5_SnI_3_ (FASI) under an Ar atmosphere at 10 K·min^–1^.

In order to further verify the presence of volatile species from the inorganic component of the perovskite below 700 K, TG-DSC measurements under similar conditions were carried out also on SnI_2_ and SnI_4_. The resulting curves, shown in Figure 3a and Figure 3b, respectively, are in agreement with previous literature data^37^ and draw attention to the much higher volatility of SnI_4_ compared to SnI_2_. The two endothermic peaks present in both DSC curves are due to the fusion and vaporization of tin iodides. Note that in the TG curve of SnI_2_ (Figure 3a), a first mass loss step is observed at a temperature similar to that corresponding to the complete sublimation of SnI_4_ (Figure 3b), most probably due to Sn­(IV) impurities in the SnI_2_ sample. The comparison with the TG curves of CN_2_H_5_SnI_3_ provides a confirmation of the previous hypotheses on the decomposition sequence of FASI: the first mass loss step can be attributed, in large part, to the release of Sn­(IV) impurities (see Section 4 for more details), while the decomposition step occurring up to about 700 K involves, along with decomposition products from the organic portion of CN_2_H_5_SnI_3_ as already observed for FAPI,^29^ the sublimation of SnI_2_ (as results also from KEMS experiments), as evident from the comparison of Figures 1 and 3a (see also Section 3.3). In conclusion, the analysis of the TG curves shows two crucial differences between FASI and FAPI, which can be related (i) to the presence of Sn­(IV) impurities in FASI, bringing about the release of the highly volatile Sn­(IV) iodide at very low temperature, and (ii) to the higher volatility of solid SnI_2_, formed from decomposition of FASI, compared to PbI_2_ formed from FAPI.

**Figure 3:**
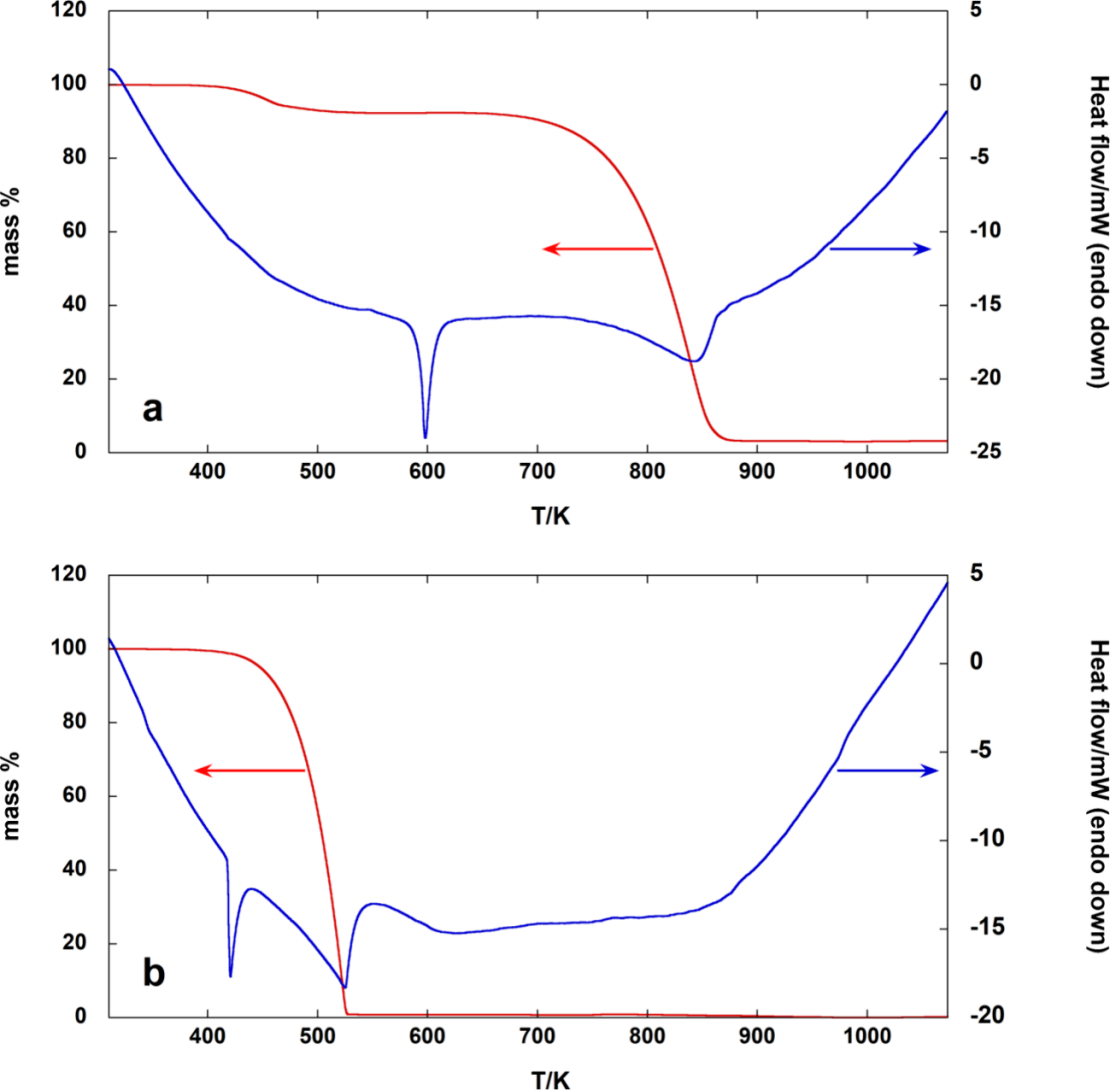
TG-DSC of (a) SnI_2_ and (b) SnI_4_ carried out under a N_2_ atmosphere (100 mL·min^–1^) at 10 K·min^–1^.

Finally, a comparison with the TG curve reported in the literature^31^ of another tin perovskite, namely, methylammonium tin triiodide (MASI), shows a similar behavior from a qualitative point of view, although with higher mass losses: a first mass loss step (around 20%) probably due to Sn­(IV) impurities can be observed for MASI from about 420 K, followed by a much more intense decrease from 620 K, leaving about 15% residual mass at 773 K.

### 3.2 Kinetic Analysis

Thermal decomposition of materials is often considered as a complex process that involves parallel and consecutive reactions. In TG experiments, the occurrence of a multistep process is usually associated with a shape change of the α vs *T* curve with varying the heating rate, where α is the degree of conversion, α (*T*) = (*m*_i_ *– m*_*T*_)/(*m*_i_ *– m*_f_), with the indices *i* and *f* representing the initial and final masses, and *m*_*T*_ the mass at a given temperature *T*. In the present study, the treatment of TG data to obtain the isoconversional temperatures at different heating rates (see the Supporting Information and Figure S1) is limited to the most significant mass loss between 550 and 700 K (Figure 1) and provides a substantial one-step α vs *T* curve at each constant heating rate, thus suggesting a simple kinetic description of the process (see below in this section), with no evident multiple-reaction effects. This evidence might seem at variance with the results of KEMS experiments (see Section 3.3 and Section 4.1), which pointed to the occurrence of three parallel decomposition processes (see eqs 9 and 11). However, the kinetic analysis was necessarily performed under conditions very different from those of KEMS. In particular, the degradation mechanism(s) can be significantly affected from effusion *vs* open pan conditions. Furthermore, as evident from Figure S1, the temperature range covered in TG experiments was much higher than that where KEMS spectra were collected. On the other hand, it cannot be excluded that the occurrence of several processes with very similar kinetic properties would result in an apparent one-step shape of α vs *T* curves.

The basic equation for the kinetic analysis is the general rate equation (GRE):

$$\frac{d\alpha }{dt}=f(\alpha )\times k(T)$$ 4

where *k*(*T*) and *f*(α) are functions depending exclusively on degree of conversion α and temperature, respectively. The temperature function is usually expressed by the Arrhenius equation *k*(*T*) = *A*·exp­(−*E*/*RT*). The reaction rate dα/d*t* is often rearranged to assume the form dα/d*T*, taking into account that TG experiments are almost exclusively performed under a linear heating rate β = d*T*/d*t*: dα/d*T* = dα/d*t ·* d*t*/d*T* = dα/d*t ·* 1/ β.

In the case of a supposed single-step process, a preliminary kinetic approach can be the integral isoconversional method denoted as Kissinger-Akahira-Sunose (KAS),^38^ based on the following relationship:

$$\ln\left(\beta /T^{2}\right)=-b\left(E/RT\right)+c$$ 5

where *b* and *c* are fitting constants independent of temperature. This equation is derived on the assumption of an approximation (Doyle’s one^39^ is applied in the KAS method) to solve a temperature integral obtained after separation of conversion and temperature variables that has no mathematical solution. As a consequence, activation energy calculated by the slope of the regression lines (one for each defined degree of conversion) must be considered almost constant^40^ to consider this method valid.

Alternatively, to have a reliable treatment of data and reasonable results that do not depend on the possible variation of activation energy with the extent of conversion, an incremental isoconversional method (IIM), proposed by Šimon and co-workers,^41^ was considered.

The usual plot of the conversion dependence of the activation energy (Figure 4) for the thermal decomposition of FASI revealed the excellent agreement of activation energies derived by KAS and IIM methods within the estimated associated uncertainties. Almost constant *E* values were obtained between about 110 and 130 kJ·mol^–1^, which are significantly lower than those obtained for FAPI by using the same method.^29^

**Figure 4:**
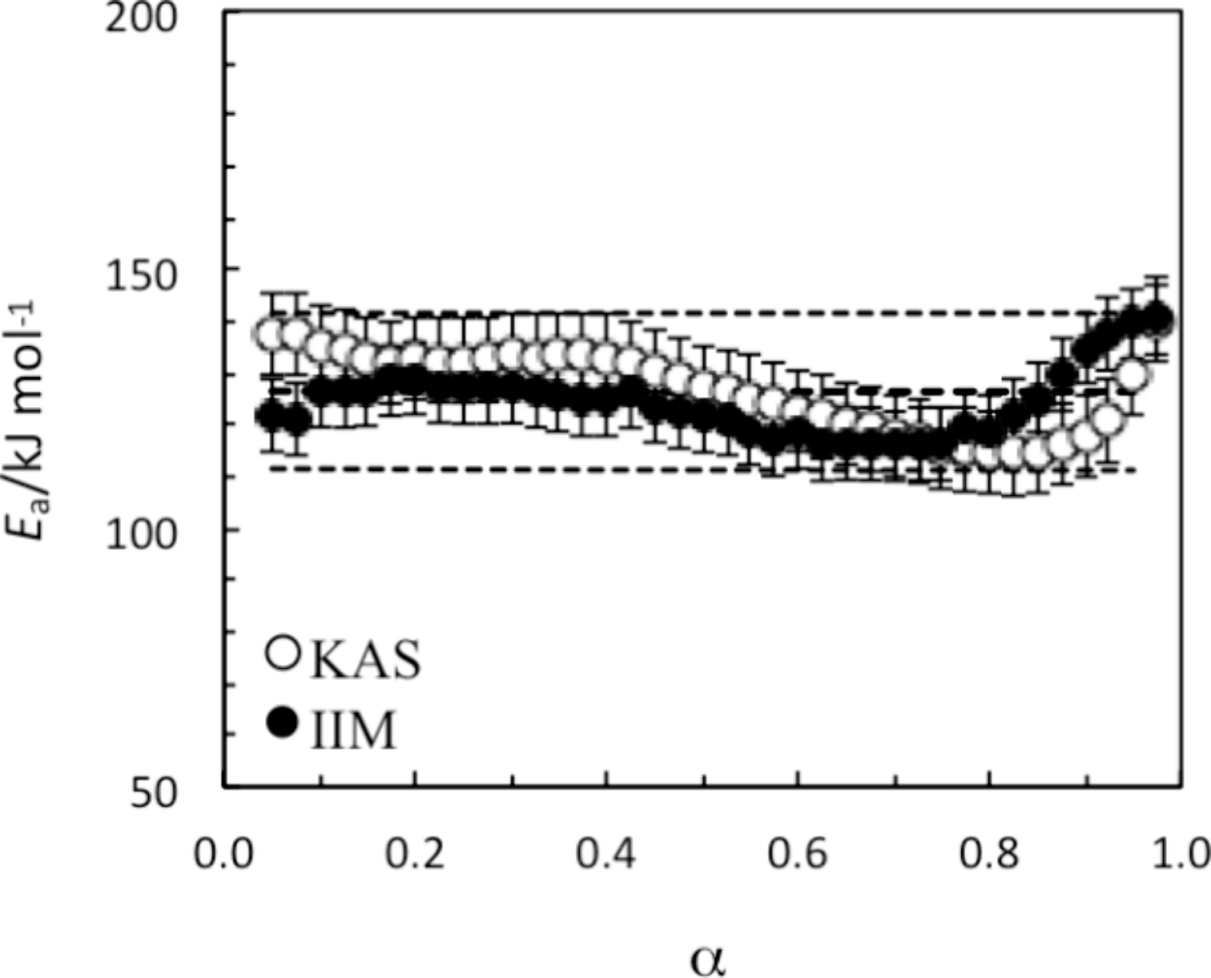
Conversion dependence of the activation energy for the thermal decomposition of FASI according to the two kinetic methods considered.

On the basis of the above results, the reaction times needed to achieve a given degree of conversion α could be estimated, similarly to what has been recently made for the thermal decomposition of some ionic liquids,^42^ using eq 6:

$$t_{\alpha }=\Sigma_{i}\Delta t_{i}=\Sigma_{i}A_{i}\times \exp(-E_{i}/RT)$$ 6

where *A*_*i*_ and *E*_*i*_ are the Arrhenius pair related to the corresponding degree of conversion. In this study, decomposition times of FASI were extrapolated to temperature values (333, 343, and 353 K) much lower compared to the experimental ones, in order to predict the thermal behavior under conditions typically involved in solar cell operation. The reaction time values t_α_ calculated from the above-described procedure for low degree of conversion (α < 0.25) are reported in Figure 5. Despite the rather crude extrapolation involved in the procedure described above, these results can be used for a rough estimate of the degree of the degradation of the light-harvesting material in FASI-containing photovoltaic cells under various temperature conditions.

**Figure 5:**
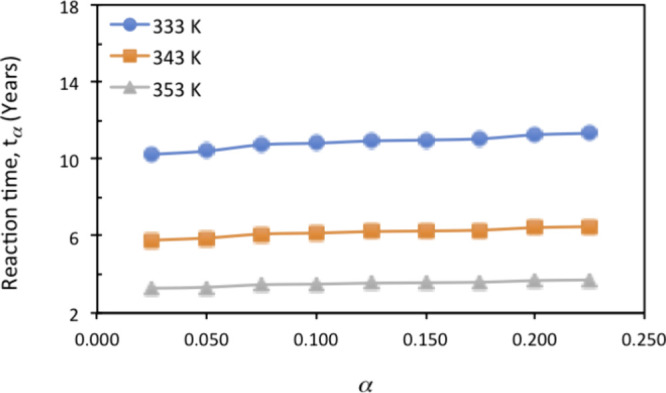
Estimated reaction time *t*_α_ for the decomposition process of CN_2_H_5_SnI_3_ (FASI) at selected temperatures (333–353 K) for low degrees of conversion.

Besides the isoconversional method based on TG measurements, the kinetic study of the nucleation and growth processes of SnI_2_ involved in the decomposition of FASI by temperature-controlled powder X-ray diffraction experiments was also attempted, by using the Johnson–Mehl–Avrami–Kolmogorov (JMAK) model, as performed in the previous work on FAPI.^29^ However, different experiments at different but very close temperatures (the useful temperature range in which the kinetics of the decomposition of FASI to SnI_2_ and gaseous products could be observed in experimentally reasonable time intervals was 513–531 K, and the temperature difference between each isothermal experiment was 2 K), gave very different values, ranging from 1 to 4, of the Avrami exponent *n* of the equation:

$$a=1-\exp\left(-{kt}^{n}\right)$$ 7

where *a* is the volume fraction of the phase SnI_2_, *k* is the kinetic constant, and *t* is time. Different values of the Avrami exponent *n* mean different mechanisms of nucleation and growth processes,^43^ which is hardly plausible when a sufficiently pure starting material is used in a very narrow temperature range and no phase transitions occur, as verified in the previous work on FAPI. The most plausible explanation lies in the fact that the atmosphere of the nonambient XRD reaction chamber^29^ cannot be evacuated, and air can be replaced only by an appropriate gas flow (He in our case); therefore, the process to obtain a reasonably oxygen-free atmosphere after inserting the sample requires many hours. With FASI being extremely prone to oxidation (Sn^2+^ to Sn^4+^), it is highly probable that the samples, when the XRD experiments were performed, contained significant amounts of Sn^4+^ that influenced in a noncontrollable and nonreproducible way the kinetics of the nucleation and growth processes involved in FASI decomposition. Consequently, the experimental data obtained by temperature-controlled powder X-ray diffraction measurements and their analysis with the JMAK model are not reported in the present work, being of no use, despite the time-consuming experimental and data analysis efforts.

### 3.3 Partial Pressures of Decomposition Products from Knudsen Effusion Mass Spectrometry

As previously mentioned, we carried out a number of experiments under effusion conditions, i.e., with the sample placed inside a Knudsen cell. The KEMS and KEML techniques (the latter limited to preliminary experiments) were used. In the first method, the effusing vapors are analyzed by a mass spectrometer, whereas in KEML, the mass of the evaporating sample is continuously monitored by a thermobalance, as detailed in Section 2.5.

Preliminary KEML experiments were aimed at verifying if an invariant value of pressure was attained when the FASI sample was heated at a given constant temperature under the effusion conditions. Indeed, a constant value of pressures would suggest the attainment of a thermodynamic heterogeneous equilibrium between the solid and gaseous phases, whereas a continuous change would indicate a corresponding change in activities of the components in the decomposing solid. The results of isothermal KEML experiments shown in Figure S2 point rather to the latter view, with vapor pressures continuously decreasing as the decomposition proceeds. This evidence also discouraged any thermodynamic analysis of partial pressure data derived from the KEMS measurements.

KEMS experiments covered the overall temperature range of 366–462 K. The complete list of the ion species observed in the KEMS spectra is reported in Table 1 with a tentative assignment of neutral precursors.

**Table 1:** List of Ion Species Detected in KEMS Spectra of CN_2_H_5_SnI_3_ with Assigned Neutral Precursors

**ion**	***m*/*z***	**neutral species**
**NH**_**2**_^**+**^	16	NH_3_
**NH**_**3**_^**+**^ **or OH** ^**+**^ ^a^	17	NH_3_ or H_2_O^a^
**H**_**2**_ **O** **^+^ ^a^** **or NH**_**4**_^**+**^	18	NH_3_ or H_2_O^a^
**CN** ^**+**^	26	HCN
**HCN** ^**+**^	27	HCN
**H**_**2**_ **CN** ^**+**^	28	C_3_N_3_H_3_ ^b^
**CH**_**2**_ **N**_**2**_^**+**^	42	CH_4_N_2_ ^c^
**CH**_**3**_ **N**_**2**_^**+**^	43	CH_4_N_2_ ^c^
**CH**_**4**_ **N**_**2**_^**+**^	44	CH_4_N_2_ ^c^
**C**_**2**_ **N**_**2**_ **H** ^**+**^	53	C_3_N_3_H_3_ ^b^
**C**_**2**_ **N**_**2**_ **H**_**2**_^**+**^	54	C_3_N_3_H_3_ ^b^
**C**_**3**_ **N**_**3**_ **H**_**3**_^**+**^	81	C_3_N_3_H_3_ ^b^
**Sn** ^**+**^	120	SnI_2_ and SnI_4_
**I** ^**+**^	127	HI, SnI_2_, and SnI_4_
**HI** ^**+**^	128	HI
**SnI** ^**+**^	247	SnI_2_ and SnI_4_
**SnI**_**2**_^**+**^	374	SnI_2_ and SnI_4_
**SnI**_**3**_^**+**^	501	SnI_4_
**SnI**_**4**_^**+**^	628	SnI_4_

a Signal at 18 *m*/*z* was found to decrease significantly during the experiment; therefore, H_2_O partial pressure was not calculated.

b Triazine.

c Formamidine.

In analyzing Table 1, it should be first of all noted that, besides species coming from the decomposition of the organic cation, already observed for FAPI,^29^ several Sn–I ions were detected in the vapor produced from FASI, indicating the simultaneous decomposition/release of the inorganic portion in the latter. This result convincingly confirms what was suggested by the TG curves discussed in Section 3.1. To the best of our knowledge, this is the first evidence of this kind of behavior in a tin-based perovskite.

Appearance energies, useful for the identification of neutral precursors of the detected species, were measured for the most intense ions and are reported in Table S1, together with ionization energies of the corresponding neutral molecules taken from ref 32, where available.

Several representative mass spectra collected in KEMS experiments are reported in Figure 6a–c. The spectra shown in the figures were recorded at different temperatures and vaporization times. Indeed, it should be noted that the observed signals were found to depend on both variables, consistently with KEML experiments (see again Figure S2). Unlike what one could expect under effusion conditions for a monovariant heterogeneous equilibrium, a stable value of the intensity was not attained at a constant temperature.

**Figure 6:**
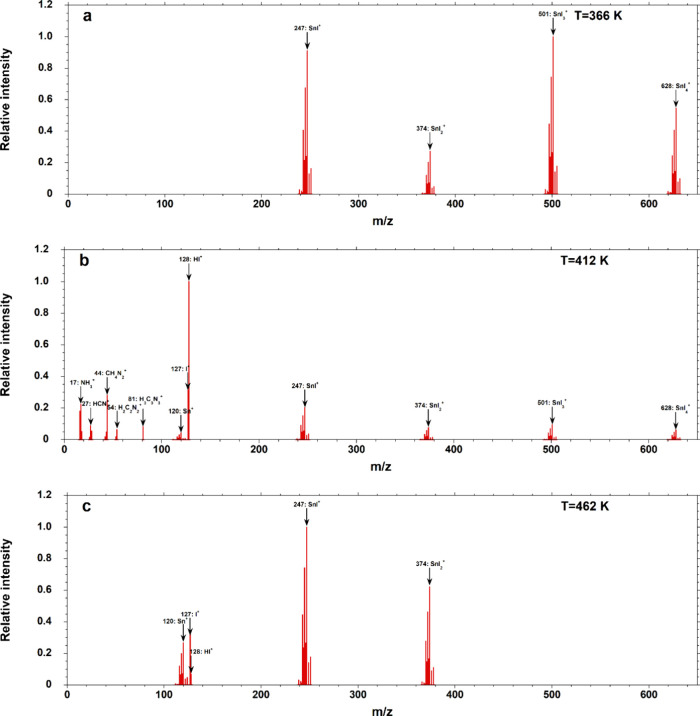
KEMS spectra of the CN_2_H_5_SnI_3_ vapors recorded at (a) 366 K, (b) 412 K, and (c) 462 K and increasingly longer vaporization times, from (a) to (c).

In particular, in the first part of any experiment on fresh samples, also corresponding to the lowest temperatures (since the experiments were carried out by gradually increasing the temperature of the effusion cell), only Sn–I ions were detected (namely, SnI^+^, SnI_2_^+^, SnI_3_^+^, and SnI_4_^+^), as shown in Figure 6a, where the spectrum at the lowest equilibrated temperature is reported (note that the same ions were already detected at temperatures as low as 318 K). In order to identify the neutral species in the gas phase at this stage, additional vaporization experiments under effusion conditions were performed on SnI_4_,^44^ at lower temperatures than previously reported studies. The mass spectrum of the vapor phase over pure SnI_4_ measured at 324 K is shown as an example in Figure S3. This spectrum revealed the presence of the same Sn–I ions as those originated from CN_2_H_5_SnI_3_ thermal decomposition, along with Sn^+^, I^+^, and I_2_^+^ (nevertheless, the actual formation of the latter two in the Knudsen cell was questionable, see ref 44). As regards the Sn^+^ ion, it is the species with the lowest relative intensity in the SnI_4_ KEMS spectrum and probably was not detected in the gas phase of the perovskite at 366 K, due to the overall lower absolute intensity of all the signals in the latter (about two orders of magnitude less than in SnI_4_). The mixed Sn–I ion species SnI^+^, SnI_2_^+^, SnI_3_^+^, and SnI_4_^+^ exhibited similar relative intensities in the two samples, although SnI_3_^+^ was the most intense peak in the CN_2_H_5_SnI_3_ spectrum at 366 K, while the SnI_4_^+^ molecular ion was slightly predominant in the SnI_4_ spectrum. This analysis led us to reasonably conclude that the only species released by CN_2_H_5_SnI_3_ at such low temperatures and at the initial times of the experiments was gaseous SnI_4_.

By increasing the temperature and as the degradation of the sample proceeded, the gas phase was enriched with species arising from the decomposition of the organic component of the perovskite, as can be seen in Figure 6b, where the spectrum recorded at 412 K is reported. This spectrum displays, along with Sn–I ions, already detected at lower temperatures, Sn^+^ (with low intensity), I^+^, HI^+^ (the most intense peak at this temperature), C_3_N_3_H_3_^+^, C_2_N_2_H_2_^+^, CH_4_N_2_^+^, HCN^+^, and NH_3_^+^, similarly to what had been observed for CN_2_H_5_PbI_3_ (although at higher temperatures).^29^ From the measured appearance energies (Table S1), C_2_N_2_H_2_^+^ was attributed to fragmentation of C_3_N_3_H_3_, while HCN^+^, which could also be a fragment of C_3_N_3_H_3_, was considered at least partly as a molecular ion since its appearance energy was found to correspond to the HCN ionization energy. Also, I^+^ resulted in a fragment, with an appearance energy almost 3 eV higher than the ionization energy of the I atom (13.2 vs 10.5 eV). This ion could arise from different neutral precursors, namely, HI, SnI_2_, and SnI_4_ (as explained in the Supporting Information), while NH_3_^+^, C_3_N_3_H_3_^+^, and CH_4_N_2_^+^ were considered as molecular ions corresponding to ammonia, triazine, and formamidine neutral precursors. The mixed Sn–I ions SnI^+^, SnI_2_^+^, SnI_3_^+^, and SnI_4_^+^ showed noticeably different relative intensities compared to those observed in the CN_2_H_5_SnI_3_ mass spectrum at 366 K (and also in the SnI_4_ KEMS spectrum in Figure S3), with SnI^+^ being the most intense peak, pointing to the likely presence of SnI_2_ as a neutral species at this temperature. In order to corroborate this hypothesis, KEMS experiments on SnI_2_ were also carried out, in the temperature range 414–449 K. The mass spectrum of the vapor phase of SnI_2_ at 449 K is reported as an example in Figure S4. In this spectrum (as well as in spectra recorded at different temperatures, which displayed no appreciable temperature dependence of the intensity pattern), SnI^+^ is indeed the most intense peak among the Sn–I mixed species. In conclusion, the similarities with the SnI_2_ fragmentation pattern and the low relative intensities of SnI_3_^+^ and SnI_4_^+^, which must be entirely attributed to the SnI_4_ neutral species, provided confirmation of the simultaneous presence of SnI_2_ and SnI_4_ in the CN_2_H_5_SnI_3_ vapor phase in the medium temperature range of our experiments.

Lastly, the spectrum reported in Figure 6c, recorded at the highest temperature of our experiments (462 K), illustrates the composition of the gas phase after prolonged vaporization times, thus when extensive decomposition of the sample had already taken place. Remarkably, it shows the disappearance of SnI_3_^+^ and SnI_4_^+^ ions, indicating the disappearance of SnI_4_ as a neutral species, as well as that of species produced by the decomposition of the organic portion, except for HI^+^, which presents, however, the lowest relative intensity in this spectrum. This led us to the conclusion that the gaseous decomposition products released by CN_2_H_5_SnI_3_ at high temperature and after significant thermal degradation are represented essentially by SnI_2_, together with very low amounts of residual HI from the organic portion. At this stage, Sn­(IV) in the solid was completely depleted, and the organic portion is in large part decomposed and volatilized.

For the calculation of partial pressures of the species produced by CN_2_H_5_SnI_3_ thermal decomposition, eq 1 was employed. The procedure used for assignment of intensity contributions to ions that could arise from more than one neutral precursor is discussed in the Supporting Information. From such considerations, it was possible to calculate partial pressures of all the species found in the gas phase produced from CN_2_H_5_SnI_3_ thermal decomposition, namely, NH_3_, HCN, CH_4_N_2_ (formamidine), C_3_N_3_H_3_ (triazine), HI, SnI_2_, and SnI_4_, in the explored temperature range. Total ionization cross sections, σ_*i*_, necessary for the application of eq 1,^44–54^ required some estimations in the case of species for which experimental or computational values were not available in the literature, and this is likely to introduce uncertainties in the obtained pressure values. Details on the estimation of σ_*i*_ values are reported in the Supporting Information. The so-determined pressure values are reported in Table S2.

## 4 Discussion

### 4.1 Decomposition Pathways of Formamidinium Tin Triiodide

Partial pressures of SnI_2_ and SnI_4_ measured in the gas phase produced upon CN_2_H_5_SnI_3_ thermal degradation (see Table S2) are compared in Figure 7a,b with those measured by vaporization of binary iodides and with thermochemical data from the literature.

**Figure 7:**
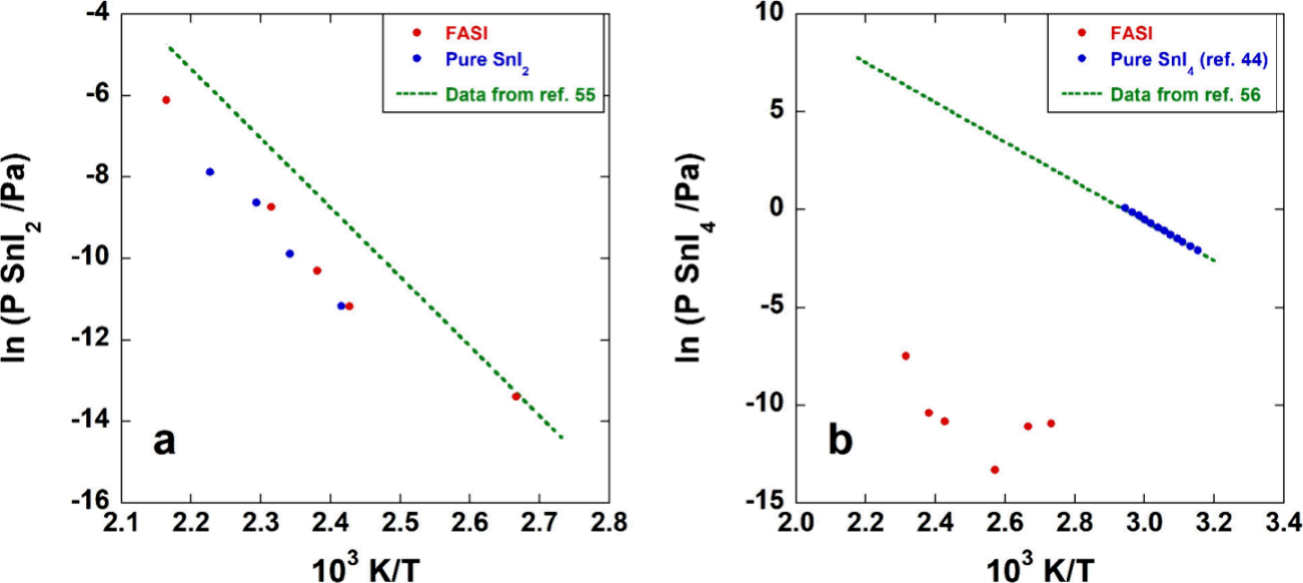
(a) SnI_2_ and (b) SnI_4_ partial pressures in the CN_2_H_5_SnI_3_ gas phase (in red) compared to the vapor pressure of pure compounds (in blue) and literature data (dashed lines).

From Figure 7a, it is possible to state that SnI_2_ is released by CN_2_H_5_SnI_3_ with a very similar vapor pressure (red symbols) to that of pure SnI_2_ (blue symbols), pointing to the presence, as a decomposition product, of the corresponding solid compound, which undergoes sublimation. We note that vapor pressure values determined in our KEMS experiments resulted to be lower by a factor about 6–7 than those found in the literature, obtained by previous measurements carried out with the same technique.^55^ Different methods used for the estimation of ionization cross-section values can partially explain this discrepancy. Conversely, as concerns SnI_4_ (see Figure 7b), partial pressures measured in the gas phase produced by the perovskite are dramatically lower than vapor pressures of pure SnI_4_ (around five orders of magnitude), which in this case are in agreement with data from the IVTANTHERMO thermochemical database.^56^ This evidence indicates that SnI_4_ is not formed as a pure phase in the solid and may be explained with a low thermodynamic activity of Sn­(IV) in the decomposing perovskite, bringing about a much lower partial pressure of SnI_4_. Since this compound is most probably produced in the gas phase as a consequence of the presence of Sn­(IV) ions “dissolved” in the perovskite structure, it can be expected that the loss rate of SnI_4_ from the solid is strongly dependent on their concentration, in turn depending on the synthesis and storage conditions. Further evidence for this picture was provided by XRD analysis of the solid residue after KEMS in comparison with the pattern of pristine CN_2_H_5_SnI_3_ and SnI_2_. As shown in Figure 8, the only crystalline phases in the residue are the perovskite and SnI_2_, with no SnI_4_ detected (incidentally, we note that this sample was not subjected to the highest temperatures of our experimental range and that a sample heated for longer times and up to 462 K revealed almost complete amorphization from XRD analysis).

**Figure 8:**
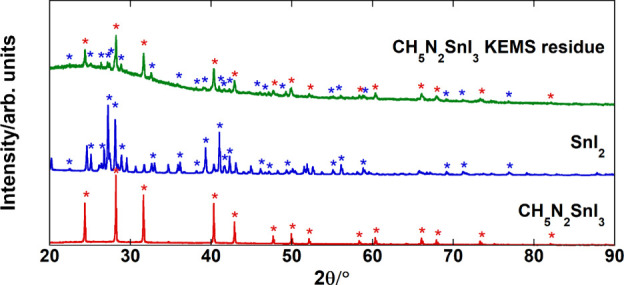
Powder XRD patterns of CN_2_H_5_SnI_3_ (in red), SnI_2_ (in blue), and the residue after KEMS experiments on CN_2_H_5_SnI_3_ up to 432 K (in green).

We can thus conclude that a first degradation channel of FASI, observed already at temperatures lower than those reached during solar cell operations, is represented by

$$\mathrm{Sn}\left(\mathrm{IV}\right)\left(\mathrm{ss}\right)+4I^{-}\to {\mathrm{SnI}}_{4}\left(g\right)$$ 8

where “ss” stands for “solid solution” (tetravalent tin dissolved in the perovskite structure). This offers further confirmation of the key role played by Sn­(II) to Sn­(IV) oxidation in the degradation of tin-based perovskites, already pointed out in ref 26. To the best of our knowledge, this is the first direct observation of its release as a gaseous decomposition product.

As concerns the species arising from the decomposition of the organic component of CN_2_H_5_SnI_3_, the analysis of their partial pressures (see previous section) allowed us to identify different gas-releasing degradation reactions, similar to those previously found to occur in CN_2_H_5_PbI_3_ ^29^:

$${\mathrm{CN}}_{2}H_{5}{\mathrm{SnI}}_{3}\left(s\right)⇌{\mathrm{SnI}}_{2}\left(s,g\right)+{\mathrm{CN}}_{2}H_{4}\left(g\right)+\mathrm{HI}\left(g\right)$$ 9

$${\mathrm{CN}}_{2}H_{5}{\mathrm{SnI}}_{3}\left(s\right)⇌{\mathrm{SnI}}_{2}\left(s,g\right)+{\mathrm{NH}}_{3}\left(g\right)+1/3H_{3}C_{3}N_{3}\left(g\right)+\mathrm{HI}\left(g\right)$$ 10

$${\mathrm{CN}}_{2}H_{5}{\mathrm{SnI}}_{3}\left(s\right)⇌{\mathrm{SnI}}_{2}\left(s,g\right)+{\mathrm{NH}}_{3}\left(g\right)+\mathrm{HCN}\left(g\right)+\mathrm{HI}\left(g\right)$$ 11

From the ratios between partial pressures of CH_4_N_2_ (formamidine) and H_3_C_3_N_3_ (triazine) and of CH_4_N_2_ and HCN, reported in Table S3, it is possible to conclude that both (9) and (10) are favored over (11), which is nonetheless not negligible, and also that (9) is slightly favored over (10) at low temperature. As already highlighted, a striking difference with FAPI, experimentally found for the first time, is represented by the fact that unlike its lead counterpart, for which PbI_2_ was found to be produced exclusively as a solid in the temperature range of KEMS experiments, also carried out at higher temperatures, gaseous SnI_2_ is released from CN_2_H_5_SnI_3_ at temperatures as low as 375 K.

## 5 Conclusions

The nature of the gaseous species released from formamidinium tin triiodide (FASI) under heating was, for the first time, investigated in a wide temperature range, in order to identify the different degradation processes of this material under real operating conditions. The thermal decomposition of FASI was found to occur through different pathways with the release of various inorganic and organic species. The first gas species to be released under heating (*T* > 318 K) was SnI_4_(g), providing evidence for the role of tetravalent tin ions/atoms in the degradation of the material under conditions similar to those normally established during photovoltaic operation. The pressure of released SnI_4_(g) was measured and compared for the first time to the vapor pressure of pure SnI_4_(s), suggesting the presence of low-activity Sn­(IV) ions dissolved in the perovskite structure. At higher temperatures (*T* > 393 K), a number of species were observed in the vapor phase under effusion conditions, indicating the co-occurrence of several decomposition processes:

$$\mathrm{Sn}\left(\mathrm{IV}\right)\left(\mathrm{ss}\right)+4I^{-}\to {\mathrm{SnI}}_{4}\left(g\right)$$

$${\mathrm{CN}}_{2}H_{5}{\mathrm{SnI}}_{3}\left(s\right)\to {\mathrm{SnI}}_{2}\left(s,g\right)+{\mathrm{CN}}_{2}H_{4}\left(g\right)+\mathrm{HI}\left(g\right)$$

$${\mathrm{CN}}_{2}H_{5}{\mathrm{SnI}}_{3}\left(s\right)\to {\mathrm{SnI}}_{2}\left(s,g\right)+{\mathrm{NH}}_{3}\left(g\right)+1/3H_{3}C_{3}N_{3}\left(g\right)+\mathrm{HI}\left(g\right)$$

$${\mathrm{CN}}_{2}H_{5}{\mathrm{SnI}}_{3}\left(s\right)\to {\mathrm{SnI}}_{2}\left(s,g\right)+{\mathrm{NH}}_{3}\left(g\right)+\mathrm{HCN}\left(g\right)+\mathrm{HI}\left(g\right)$$

with the latter process apparently less important compared to the second and third ones. The most abundant species in the vapor phase was found to be HI­(g), which is formed in all of the decomposition processes.

The observed behavior is very different from that found in the lead-containing analogue (FAPI), in that the decomposition temperature is significantly lowered for the presence of tetravalent tin, and the vapor phase contains, besides the organic fragments coming from the decomposition of the cationic portion of the compound, also inorganic (tin iodides) species. For the first time, the release of these species in the gas phase from FASI under heating at moderate temperatures was observed. The much higher volatility of Sn­(IV) compared to Sn­(II) iodide and of SnI_2_ compared to PbI_2_ makes the thermal degradation pattern of FASI very different from that of FAPI, with important consequences on the feasibility of photovoltaic applications. In order to get a rough estimate of the lifetime of the material under realistic operation temperatures (333 to 353 K), we also performed a kinetic analysis based on thermogravimetry measurements by using the integral and incremental isoconversional methods.

## Supplementary Material



## Notes

Details of the kinetic analysis; temperature dependence of the degree of conversion for the decomposition of FASI at different heating rates (Figure S1); mass loss rate measured by KEML at 363 and 393 K (Figure S2); appearance potentials of the ions detected by KEMS (Table S1); KEMS spectra of pure SnI_4_ and SnI_2_ (Figures S3 and S4); details on the procedures for assignment of neutral precursors of ions in KEMS experiments and for estimation of ionization cross sections; partial pressures of species detected in FASI gas phase (Table S2); and partial pressure ratios between FASI decomposition products (Table S3) (PDF)

## References

[1] Renewable Energy Consumption https://www.iea.org/reports/renewables-2024/global-overview#abstract. (accessed Feb 11, 2025).

[2] Pastuszak J., Węgierek P. (2022). Photovoltaic
Cell Generations and Current Research
Directions for Their Development. Materials.

[3] Kojima A., Teshima K., Shirai Y., Miyasaka T. (2009). Organometal Halide
Perovskites as Visible-Light Sensitizers for Photovoltaic Cells. J. Am. Chem. Soc..

[4] Green M., Ho-Baillie A., Snaith H. (2014). The emergence of perovskite solar
cells. Nat. Photonics.

[5] Chouhan L., Ghimire S., Subrahmanyam C., Miyasaka T., Biju V. (2020). Synthesis,
optoelectronic properties and applications of halide perovskites. Chem. Soc. Rev..

[6] Chowdhury T.
A., Bin Zafar M. A., Sajjad-Ul Islam M., Shahinuzzaman M., Islam M. A., Khandaker M. U. (2023). Stability
of perovskite solar cells:
issues and prospects. RSC Adv..

[7] Kim H. J., Han G. S., Jung H. S. (2024). Managing the lifecycle of perovskite
solar cells: Addressing stability and environmental concerns from
utilization to end-of-life. eScience.

[8] Chen C.-H., Cheng S.-N., Cheng L., Wang Z.-K., Liao L.-S. (2023). Toxicity,
Leakage, and Recycling of Lead in Perovskite Photovoltaics. Adv. Energy Mater..

[9] Wang Y., Liu J., Liu Y., Li S., Xu X., Lou Z. (2024). Recent advances
in lead-free halide perovskites: from synthesis to applications. J. Mater. Chem. C.

[10] Green M. A., Dunlop E. D., Yoshita M., Kopidakis M., Bothe K., Siefer G., Hao X., Jiang J. Y. (2025). Solar Cell
Efficiency Tables (Version 65). Prog. Photovolt.
Res. Appl..

[11] Shi Y., Zhu Z., Miao D., Ding Y., Mi Q. (2024). Interfacial Dipoles
Boost Open-Circuit Voltage of Tin Halide Perovskite Solar Cells. ACS Energy Lett..

[12] Hu W., He X., Fang Z., Lian W., Shang Y., Li X., Zhou W., Zhang M., Chen T., Lu Y. (2020). Bulk heterojunction
gifts bismuth-based lead-free perovskite solar
cells with record efficiency. Nano Energy.

[13] Akbulatov A. F., Frolova L. A., Dremova N. N., Zhidkov I., Martynenko V. M., Tsarev S. A., Luchkin S. Y., Kurmaev E. Z., Aldoshin S. M., Stevenson K. J. (2020). Light or Heat: What Is Killing Lead Halide
Perovskites under Solar Cell Operation Conditions?. J. Phys. Chem. Lett..

[14] Ma L., Guo D., Li M., Wang C., Zhou Z., Zhao X., Zhang F., Ao Z., Nie Z. (2019). Temperature-Dependent
Thermal Decomposition Pathway of Organic–Inorganic Halide Perovskite
Materials. Chem. Mater..

[15] Brunetti B., Cavallo C., Ciccioli A., Gigli G., Latini A. (2016). On the Thermal
and Thermodynamic (In)­Stability of Methylammonium Lead Halide Perovskites. Sci. Rep..

[16] Latini A., Gigli G., Ciccioli A. (2017). A study on
the nature of the thermal
decomposition of methylammonium lead iodide perovskite, CH_3_NH_3_PbI_3_: an attempt to rationalise contradictory
experimental results. Sustainable Energy Fuels.

[17] Ciccioli A., Latini A. (2018). Thermodynamics and
the Intrinsic Stability of Lead
Halide Perovskites CH_3_NH_3_PbX_3_. J. Phys. Chem. Lett..

[18] Burwig T., Pistor P. (2021). Reaction kinetics of
the thermal decomposition of MAPbI_3_ thin films. Phys. Rev. Mater..

[19] Burwig T., Heinze K., Pistor P. (2022). Thermal decomposition
kinetics of
FAPbI_3_ thin films. Phys. Rev. Mater..

[20] Yang W.-F., Igbari F., Lou Y.-H., Wang Z.-K., Liao L.-S. (2020). Tin Halide
Perovskites: Progress and Challenges. Adv. Energy
Mater..

[21] Seo J., Song T., Rasool S., Park S., Kim J. Y. (2023). An Overview
of Lead, Tin, and Mixed Tin–Lead-Based ABI_3_ Perovskite
Solar Cells. Adv. Energy Sustainability Res..

[22] Zhang Z., Wang L., Bi H., Baranwal A. K., Kapil G., Sanehira Y., Liu J., Liu D., Shen Q., Hayase S. (2024). Enhancement of Efficiency and Stability
for Tin Halide
Perovskite Solar Cells by Using Improved Doping Method. Adv. Optical Mater..

[23] Galve-Lahoz S., Sánchez-Diaz J., Echeverría-Arrondo C., Simancas J., Rodriguez-Pereira J., Turren-Cruz S.-H., Martinez-Pastor J. P., Mora-Seró I., Delgado J. L. (2024). Addressing ambient stability challenges
in pure FASnI_3_ perovskite solar cells through organic additive
engineering. J. Mater. Chem. A.

[24] Chen L., Fu S., Li Y., Sun N., Yan Y., Song Z. (2024). On the Durability
of Tin-Containing Perovskite Solar Cells. Adv.
Sci..

[25] Akbulatov A. F., Tsarev S. A., Elshobaki M., Luchkin S. Y., Zhidkov I., Kurmaev E. Z., Aldoshin S. M., Stevenson K. J., Troshin P. A. (2019). Comparative Intrinsic Thermal and
Photochemical Stability
of Sn­(II) Complex Halides as Next-Generation Materials for Lead-Free
Perovskite Solar Cells. J. Phys. Chem. C.

[26] Lanzetta L., Webb T., Zibouche N., Liang X., Ding D., Min G., Westbrook R. J. E., Gaggio B., Macdonald T. J., Islam M. S. (2021). Degradation mechanism of hybrid tin-based perovskite
solar cells and the critical role of tin (IV) iodide. Nat. Commun..

[27] Webb T., Haque S. A. (2024). A comparison of
molecular iodine evolution on the chemistry
of lead and tin perovskites. Energy Environ.
Sci..

[28] Li B., Jayawardena K. D. G. I., Zhang J., Bandara R. M. I., Liu X., Bi J., Silva S. M., Liu D., Underwood C. C. L., Xiang Y., Ma Y., Zhang W., Silva S. R. P. (2024). Stability
of formamidinium tin triiodide-based inverted perovskite solar cells. Renew. Sust. Energy Rev..

[29] Luongo A., Brunetti B., Vecchio
Ciprioti S., Ciccioli A., Latini A. (2021). Thermodynamic
and Kinetic Aspects of Formamidinium Lead Iodide Thermal Decomposition. J. Phys. Chem. C.

[30] Howie R. A., Moser W., Trevena I. C. (1972). The crystal
structure of tin­(II)
iodide. Acta Crystallogr..

[31] Stoumpos C. C., Malliakas C. D., Kanatzidis G. D. (2013). Semiconducting Tin and Lead Iodide
Perovskites with Organic Cations: Phase Transitions, High Mobilities,
and Near-Infrared Photoluminescent Properties. Inorg. Chem..

[32] Linstrom, P. J. ; Mallard, W. G. , Eds.; NIST Chemistry WebBook, NIST Standard Reference Database Number 69; National Institute of Standards and Technology: Gaithersburg MD, 20899. 10.18434/T4D303.

[33] Brunetti B., Ciccioli A., Gigli G., Lapi A., Misceo N., Tanzi L., Vecchio Ciprioti S. (2014). Vaporization of the prototypical
ionic liquid BMImNTf_2_ under equilibrium conditions: a multitechnique
study. Phys. Chem. Chem. Phys..

[34] Mitzi D. B., Liang K. (1997). Synthesis, Resistivity,
and Thermal Properties of the Cubic Perovskite
NH_2_CH = NH_2_SnI_3_and Related Systems. J. Solid State Chem..

[35] Dang Y., Zhou Y., Liu X., Ju D., Xia S., Xia H., Tao X. (2016). Formation of Hybrid
Perovskite Tin Iodide Single Crystals
by Top-Seeded Solution Growth. Angew. Chem.,
Int. Ed..

[36] Leijtens T., Prasanna R., Gold-Parker A., Toney M. F., McGehee M. D. (2017). Mechanism
of Tin Oxidation and Stabilization by Lead Substitution in Tin Halide
Perovskites. ACS Energy Lett..

[37] Sawada Y., Suzuki M. (1994). Thermal change of SnI2
thin films: Part 1. Thermogravimetry. Thermochimica
Acta.

[38] Akahira T., Sunose T. T. (1971). Joint Convention of Four Electrical
Institutes. Res. Report Chiba Inst. Technol.
(Sci. Technol.).

[39] Doyle C. D. (1962). Estimating
isothermal life from thermogravimetric data. J. Appl. Polym. Sci..

[40] Simon P., Dubaj T., Cibulková Z. (2022). Frequent flaws
encountered in the
manuscripts of kinetic papers. J. Therm. Anal.
Calorim..

[41] Šimon P., Thomas P. S., Okuliar J., Ray A. S. (2003). An incremental integral
isoconversional method. J. Therm. Anal. Calorim..

[42] Ferdeghini C., Guazzelli L., Pomelli C. S., Ciccioli A., Brunetti B., Mezzetta A., Vecchio Ciprioti S. (2021). Synthesis, thermal behavior and kinetic
study of N-morpholinium dicationic ionic liquids by thermogravimetry. J. Mol. Liq..

[43] Shirzad K., Viney C. (2023). A critical review on
applications of the Avrami equation beyond materials
science. J. R. Soc. Interface.

[44] Romagnoli L., Almeida A. R. R. P., Silva Ferraz J. M., Latini A., Freitas V. L. S., Ribeiro
da Silva M. D. M. C., Schiavi P. G., Vecchio
Ciprioti S., Ciccioli A. (2024). Thermodynamic study of tin tetraiodide
(SnI_4_) sublimation by effusion techniques. J. Chem. Thermodyn..

[45] Bull J. N., Harland P. W., Vallance C. (2012). Absolute Total Electron
Impact Ionization
Cross-Sections for Many-Atom Organic and Halocarbon Species. J. Phys. Chem. A.

[46] Hilpert K. (1991). High temperature
mass spectrometry in materials research. Rapid
Commun. Mass. Spectrom..

[47] Drowart J., Chatillon C., Hastie J., Bonnell D. (2005). High-temperature mass
spectrometry: Instrumental techniques, ionization cross-sections,
pressure measurements, and thermodynamic data (IUPAC Technical Report). Pure Appl. Chem..

[48] Pandey A. N., Bigotto A., Gulati R. K. (1991). Quantum mechanical studies of bond
and molecular polarizabilities of gas-phase metal halides. Acta Phys. Polym., A.

[49] Kalugina Y. N., Thakkar A. J. (2015). Electric properties of stannous and
stannic halides:
How good are the experimental values?. Chem.
Phys. Lett..

[50] NIST. Standard Reference Database 107; https://www.nist.gov/pml/electron-impact-cross-sections-ionization-and-excitationdatabase, 10.18434/T4KK5C (accessed Feb 11, 2025).

[51] Vinodkumar M., Dave R., Bhutadia H., Antony B. K. (2010). Electron impact
total ionization cross sections for halogens and their hydrides. Int. J. Mass Spectrom..

[52] Pandya S. H., Shelat F. A., Joshipura K. N., Vaishnav B. G. (2012). Electron ionization
of exotic molecular targets CN, C_2_N_2_, HCN, HNC
and BF—Theoretical cross sections. Int.
J. Mass Spectrom..

[53] Gupta D., Choi H., Singh S., Modak P., Antony B., Kwon D.-C., Song M.-Y., Yoon J.-S. (2019). Total ionization
cross section of cyclic organic molecules. J.
Chem. Phys..

[54] Bull J. N., Harland P. W. (2008). Absolute electron
impact ionization cross-sections
and polarisability volumes for C2 to C4 aldehydes, C4 and C6 symmetric
ethers and C3 to C6 ketones. Int. J. Mass Spectrom..

[55] Hilpert K., Bencivenni L., Saha B. (1985). Molecular Composition
and Thermochemistry
of Tin­(II), Lead­(II), and Scandium­(III) Iodide Vapours. Ber. Bunsenges. Phys. Chem..

[56] Iorish, V. S. IVTANTHERMO database-Version 3.0; Glushko Thermocenter ofRussian Academy of Sciences, 2005.

