# Supplementary material for: Stability of Tin-Containing Hybrid Perovskites: The Thermal Decomposition of Formamidinium Tin Triiodide (FASnI3) Investigated by Thermogravimetry and Effusion Techniques
Source: J Phys Chem C Nanomater Interfaces. 2025 May 10;129(20):9291–301. doi: 10.1021/acs.jpcc.5c01854 (PMC12105013; doi:10.1021/acs.jpcc.5c01854)
Supplement: Supplementary file 1 [file jp5c01854_si_001.pdf]

Supplementary material

Stability of Tin-Containing Hybrid Perovskites: The Thermal Decomposition of Formamidinium Tin Triiodide ($FASnI_3$) Investigated by Thermogravimetry and Effusion Techniques

Martina Pesci[1], Lorenza Romagnoli*[1], Bruno Brunetti[2], Stefano Vecchio Ciprioti[3], Andrea Ciccioli*[1], Alessandro Latini*[1]

[1]Dipartimento di Chimica, Sapienza University of Rome, P.le A. Moro 5, 00185 Rome, Italy.
[2]Istituto per lo Studio dei Materiali Nanostrutturati, Consiglio Nazionale delle Ricerche, Dipartimento di Chimica, Sapienza University of Rome, P.le A. Moro 5, 00185 Rome, Italy.
[3]Dipartimento di Scienze di Base ed Applicate per l'Ingegneria (S.B.A.I.), Sapienza University of Rome, Via del Castro Laurenziano 7, Building RM017, 00161 Rome, Italy.

1. Kinetic Analysis from TG-DTA experiments at different heating rates

To carry out a kinetic analysis of thermal decomposition of FASI, experimental TG data have been processed. From each TG/DTA experiment (among the five carried out at 2, 3, 4, 7 and 10 $K \cdot min^{-1}$) the degree of conversion at each fixed temperature for the temperature range in which the decomposition process of FASI occurs has been determined according to the following expression:

$$\alpha(T) = (m_i - m_T)/(m_i - m_f), \qquad S(1)$$

where the indices *i* and *f* represent the initial and final masses, while m_T is the mass at a given temperature *T*. Fig. S1 shows the trend of the five α *vs. T* plots at each fixed heating rate, in which no change in the shape of the curves is observed but a shift toward higher temperature with increasing the heating rate. This suggested to process the data under the hypothesis of one single reaction mechanism for the decomposition process of FASI (see text for further discussion). The isoconversional analysis requires to determine the isoconversional temperatures corresponding to fixed degree of conversion at each experiment at constant heating rate.

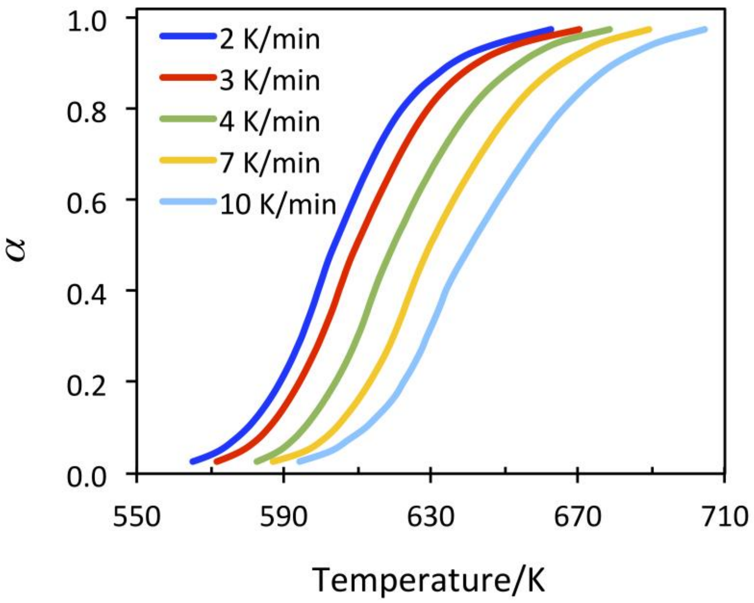

Figure S1. Temperature dependence of the degree of conversion α for the decomposition process of $CN_2H_5SnI_3$ (FASI) (see for example Fig. 2 in the text in the temperature range from about 593 to 703 K at 10 $K{\cdot}min^{-1}$) for all the TG/DTA experiments carried out at five different heating rates (2 – 10 $K{\cdot}min^{-1}$).

2. Knudsen effusion mass loss and Knudsen effusion mass spectrometry results

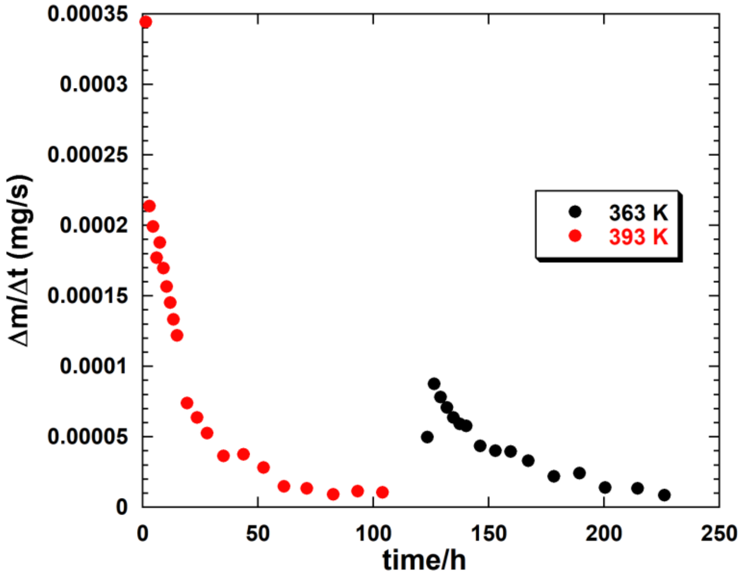

Figure S2. Isothermal mass loss rate measured by KEML for a sample of FASI consecutively at 363 K and 393 K.

Table S1. Appearance energies of the most intense ions in KEMS spectra of $CN_2H_5SnI_3$ and first ionization energies of neutral species from ref. [32].

Ion (m/z)	Appearance energy/eV [a]	Ionization energy from [32]
**NH_3^+ (17)**	9.9	10.07 ± 0.02
**HCN^+ (27)**	13.6	13.60 ± 0.01
**$CH_4N_2^+$ (44)**	9.3	-
**$C_2N_2H_2^+$ (54)**	12.2	-
**$C_3N_3H_3^+$ (81)**	10.3	9.8 - 10.1
**I^+ (127)**	13.2	10.45126
**HI^+ (128)**	9.6	10.386 ± 0.001
**SnI^+ (247)**	12.3	-
**SnI_2^+ (374)**	10.0	8.8 - 9.8
**SnI_3^+ (501)**	10.4	-
**SnI_4^+ (628)**	10.0	9.45

[a] All the measured appearance energies are given with an uncertainty of ± 0.2 eV and were assessed at *T* = 421 K.

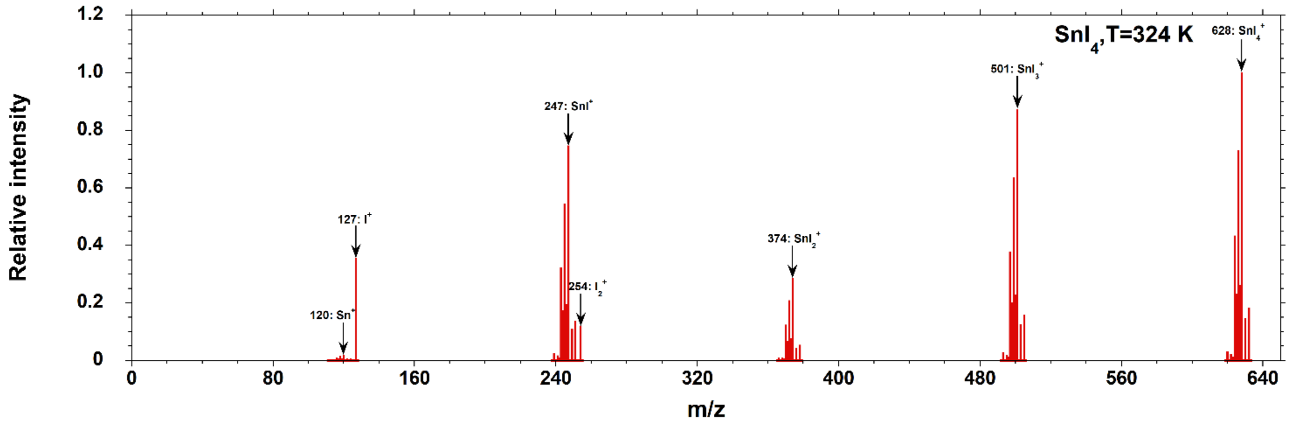

Figure S3. Mass spectrum of SnI_4 (pure tin tetraiodide) vapor phase at 324 K.

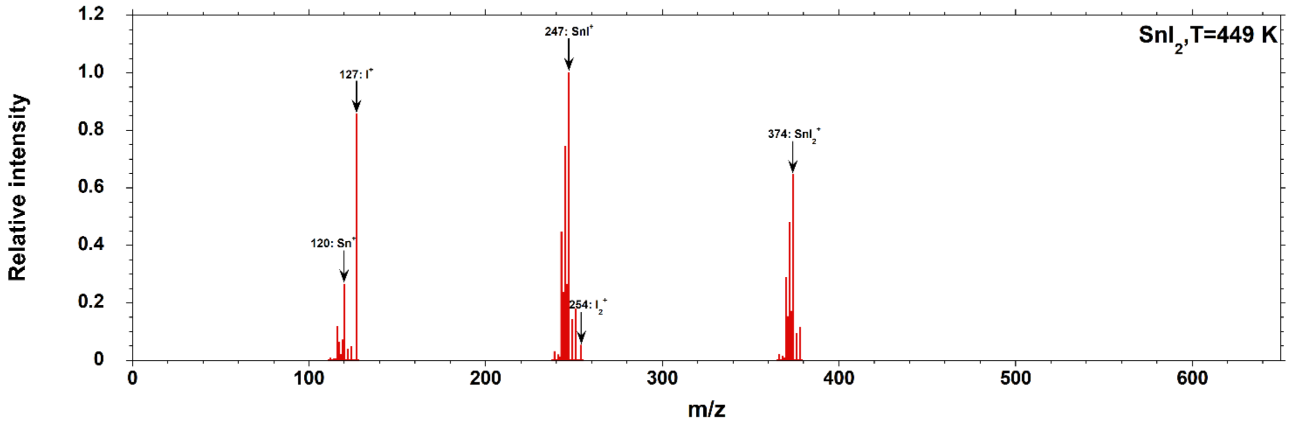

Figure S4. Mass spectrum of SnI_2 (pure tin diiodide) vapor phase at 449 K.

2.1 Assignment of neutral precursors of ions detected in KEMS experiments

Partitioning of ion intensity contributions which could arise from different neutral species was carried out based on the ratios between fragments and molecular ions in SnI_4 and SnI_2 mass spectra (Fig. S3 and S4). Thus, 30% of SnI_4^+ intensity (being the mean $\frac{I_{SnI_2^+}}{I_{SnI_4^+}}$ ratio, see [44]) was subtracted to SnI_2^+ and added to SnI_4^+, yielding SnI_2^+ intensities from neutral SnI_2 and SnI_4, respectively, while contributions of SnI_2 fragments SnI^+, Sn^+ and I^+ due to neutral SnI_2 were determined by multiplying intensities of these peaks in $CN_2H_5SnI_3$ spectra by the mean ratios between fragments and molecular ion (1.5 ± 0.2 for $\frac{I_{SnI^+}}{I_{SnI_2^+}}$, 0.45 ± 0.03 for $\frac{I_{Sn^+}}{I_{SnI_2^+}}$, 1.5± 0.1 for $\frac{I_{I^+}}{I_{SnI_2^+}}$). In turn, SnI^+ and Sn^+ portions arising from neutral SnI_4 were obtained by subtracting to total ion intensities the fractions coming from SnI_2, estimated as just illustrated, while I^+ contributions from SnI_4 were assumed to be 30% of SnI_4^+ intensity, based on intensity ratio. Finally, the remaining part of I^+ ion intensity was attributed to HI neutral precursor.

As for the ions produced upon decomposition of the organic component of the perovskite, while, as already pointed out, $C_2N_2H_2^+$ (*m/z* = 54) could be reasonably considered as $C_3N_3H_3$ fragment, based both on its higher appearance energy compared to $C_3N_3H_3^+$ (*m/z* = 81) and on $\frac{I_{54}}{I_{81}}$, being 0.79, as in the spectrum reported in ref. [32], and showing no appreciable variations with temperature, part of HCN^+ ion intensity was most probably attributable to the presence of neutral HCN in the gas phase. This portion was estimated also by comparison with $C_3N_3H_3$ spectrum from [32], in which HCN^+ fragment (*m/z* = 27) has 40 % relative intensity of the molecular ion: this percentage of total HCN^+ intensity was therefore subtracted to this ion and added to $C_3N_3H_3^+$ intensity, being attributed to $C_3N_3H_3$ fragmentation.

2.2 Electron impact ionization cross sections

In order to estimate the partial pressures from KEMS spectra by Eq (1), the values of ionization cross sections are needed.

For SnI_4, the σ value of 34 $Å^2$, as estimated in [44], was used and, by similar considerations, a cross section of 23 $Å^2$ was derived for SnI_2. This value was obtained as the mean value of results from the linear correlation between ionization cross section and polarizability, proposed in [45], and the additivity rule [46], that is, from application of Equation S(2), where *σ(M)* is the molecular total ionization cross section and *σ(A)* are the atomic ones.

$$\sigma(M) = 0.75 \cdot \sum \sigma(A) \qquad \text{S(2)}$$

Atomic ionization cross sections, whose values are 9.77 $Å^2$ and 6.03 $Å^2$ for Sn and I, respectively, were taken from [47], and by application of the above equation σ_{SnI_2}= 16.4 $Å^2$ was found, while two different values for SnI_2 polarizability from *ab initio* calculations, 16.3 $Å^3$and 18.6 $Å^3$, were found in [48] and [49], yielding ionization cross sections of 23.8 $Å^2$and 27.5 $Å^2$.

As regards the other species, 3.1 $Å^2$ as ionization cross section of NH_3 was taken from [50], while a σ of 6.47 $Å^2$ for HI was found in [51], and a value of 3.55 $Å^2$ from [52] was used for HCN. For $C_3N_3H_3$ (triazine), whose ionization cross section is not known, the value of 12 $Å^2$ was employed, estimated based on σ values of 15 $Å^2$ from [50] for benzene, values of 13.3 $Å^2$ and 15.9 $Å^2$ (obtained using different computational methods) for pyridine, found in [53], and $3 \cdot \sigma_{HCN}$ = 10.6 $Å^2$. Finally, σ of CH_4N_2 was estimated from values found in [50] for vinyloxy radical and formaldehyde, of 6.5 $Å^2$ and 4.2 $Å^2$, respectively, and that for acetaldehyde, of 6.7 $Å^2$, found in [54]. Note that all the σ values taken from the literature correspond to the maximum ionization cross section.

Table S2. Partial pressures (in Pa) of species detected in the $CN_2H_5SnI_3$ gas phase. All the values are given with an uncertainty of 50%.

T/K	*P(SnI₄)*	*P(SnI₂)*	*P(NH₃)*	*P(HCN)*	*P(CH₄N₂)*	*P(C₃H₃N₃)*	*P(HI)*
**366**	$2 \cdot 10^{-5}$	-	-	-	-	-	-
**375**	$2 \cdot 10^{-5}$	$2 \cdot 10^{-6}$	$2 \cdot 10^{-5}$	-	-	$1 \cdot 10^{-5}$	-
**389**	$2 \cdot 10^{-6}$	$5 \cdot 10^{-5}$	$2 \cdot 10^{-5}$	$5 \cdot 10^{-6}$	$4 \cdot 10^{-5}$	$3 \cdot 10^{-6}$	$1 \cdot 10^{-4}$
**412**	$2 \cdot 10^{-5}$	$1 \cdot 10^{-5}$	$2 \cdot 10^{-5}$	$3 \cdot 10^{-6}$	$1 \cdot 10^{-5}$	$5 \cdot 10^{-6}$	$6 \cdot 10^{-5}$
**420**	$3 \cdot 10^{-5}$	$3 \cdot 10^{-5}$	$3 \cdot 10^{-5}$	$1 \cdot 10^{-5}$	$3 \cdot 10^{-5}$	$9 \cdot 10^{-6}$	$2 \cdot 10^{-4}$
**432**	$6 \cdot 10^{-4}$	$2 \cdot 10^{-4}$	$2 \cdot 10^{-4}$	$7 \cdot 10^{-5}$	$1 \cdot 10^{-4}$	$4 \cdot 10^{-5}$	$2 \cdot 10^{-4}$
**462**	-	$2 \cdot 10^{-3}$	-	-	-	-	$6 \cdot 10^{-5}$

Table S3. Partial pressure ratios between decomposition products of $CN_2H_5SnI_3$ organic portion.

T/K	*$P(CH_4N_2)/P(C_3H_3N_3)$*	*$P(CH_4N_2)/P(HCN)$*
**389**	13	7.7
**412**	1.5	3.9
**420**	3.3	3.1
**432**	3.3	1.9
